# Exploring the Use and Appeal of Playpens to Protect Infants from Exposure to Animals, Animal Feces, and Dirt in Rural Ethiopia

**DOI:** 10.4269/ajtmh.20-0445

**Published:** 2020-12-02

**Authors:** Julia Rosenbaum, Eskindir Tenaw, Ron Clemmer, Morris Israel, Jeff Albert

**Affiliations:** 1 FHI 360, Washington, District of Columbia;; 2 Independent Consultant, Addis Ababa, Ethiopia;; 3 Tetra Tech, Arlington, Virginia;; 4 Aquaya Institute, San Anselmo, California

## Abstract

The persistence and pervasiveness of growth stunting in low- and middle-income countries spur reexamination of disease transmission pathways related to water, sanitation, and hygiene. Animal feces constitute a more important reservoir of enteric pathogens in homes in low-income countries than previously recognized, and exploratory object mouthing and direct ingestion of soil and animal feces represent underexplored exposure pathways. The effectiveness, adoption, constraints, and scale-up potential of measures for reducing infant and young children’s exposure to fecal pathogens are recently beginning to be systematically explored. This mixed methods study tested the feasibility and appeal of using playpens to establish a hygienic “safe zone” for infants in rural Ethiopia. We conducted home trials of three playpen designs, including two models made from locally available materials through user-centered design. After using playpens for several weeks, caregivers reported extensive benefits, ranging from perceived safety to developmental and hygiene benefits for infants and relief from physical stress and worry for caregivers. We observed many playpens contaminated with *Escherichia coli* after weeks of use, though at concentrations below those of the common room floor on which infants might otherwise have played. Caregivers reported daily playpen use, but for intervals likely insufficient to protect infants from pathogen exposure affecting growth. We determine that playpens alone cannot plausibly protect infants from environmental contamination, but our results support further exploration of the potential benefits and commercial viability of scaling up use of playpens in rural, agricultural households as part of a comprehensive approach to child development and women’s empowerment.

## INTRODUCTION

Chronic malnutrition in infants and young children (IYC), characterized by growth stunting or low height-for-age, has short- and long-term consequences for health, cognitive and motor development, learning capacity, productivity, wages, and reproductive health.^1^ Growth stunting affects as many as 165 million children younger than 5 years in low- and middle-income countries.^1^ Its pervasiveness, despite decades of nutrition and water, sanitation, and hygiene (WASH) interventions, has inspired a reexamination of WASH-related disease transmission pathways.^2^

Wagner and Lanoix’s seminal “F-diagram” has served for decades to depict routes of pathogen transmission from uncontained human feces to a new host via fluids, fields (floors, earth, and dirt), flies, fingers, food, and fomites (surfaces/objects).^3^ The classic diagram focuses exclusively on human excreta, tracing the transmission of pathogens ingested through different routes of exposure to contaminated water and food and further illustrating how WASH interventions such as sanitation, improved water quality, and hand hygiene are designed as barriers to fecal pathogen transmission.^4^

The F-diagram’s original focus was on the relationship between *human* feces and disease, but animal feces have been shown to contain enteric pathogens that contribute significantly to the global burden of disease in humans in low-income countries, particularly in rural areas.^5^ Several studies have documented significant sources of animal fecal contamination in domestic environments.^6–10^ Overnight corralling of poultry or livestock in the same room as IYC is associated with elevated markers of environmental enteric dysfunction,^11^ diarrhea, and stunting.^10,11^ Recent literature also points to exploratory mouthing of fecally contaminated objects and direct ingestion of animal feces and soil (geophagy) playing a larger role in transmission of disease to IYC than was previously recognized. Infants and young children ingest dirt and feces through mouthing, soiled fingers, play objects, and household items, as well as through exploratory ingestion of contaminated soil or poultry feces.^10–13^

It is important to note, however, that these impacts to IYC health may co-occur with potential nutritional benefits offered by improved access to food and increased household income from domestic animal husbandry.^14–16^ With their distinctly mixed findings from a cross-sectional meta-analysis of Demographic and Health Survey (DHS) data from 30 sub-Saharan African countries, Kaur et al.^14^ reinforce the hypothesis that domestic animal husbandry is, at the same time, “protective against stunting, an indicator of chronic malnutrition, and a risk factor for all-cause mortality in children.”

Taking into account these previously underexplored sources and pathways of transmission, household WASH interventions limited to sanitation, water quality improvement, and hand hygiene are likely inadequate in blocking fecal pathogen transmission, particularly to IYC.^4^ Risks from animal waste in the home environment must be addressed, but the effectiveness, adoption, constraints, and scale-up potential of measures for reducing IYC exposure, such as playmats and playpens, improved flooring, and modified animal husbandry, have only been recently systematically explored and documented.^9,10,17–24^ Three previous playpen studies^23,25,26^ and a fourth under review (S. Budge, personal communication) have assessed various aspects of feasibility, acceptability, use, and reported impact.

To contribute to this growing evidence base, we sought to determine the feasibility and acceptability of using an infant playpen to establish a hygienic “safe zone” for infants in rural Ethiopia. The specific objectives of this research were to:

1. assess caregiver use of playpens to separate infants from animals, dirt, and feces;
2. explore the feasibility and appeal of using and cleaning playpens;
3. identify playpen attributes affecting use, appeal, and perceived value; and
4. gain a better understanding of the financial value of playpens to end-users.

## METHODS AND MATERIALS

### Study overview.

We randomly selected 31 households with an infant aged 7–12 months and a caregiver aged 18 years or older across 10 purposively selected villages (*gotts*) in two wards (*kebeles*) in Bahir Dar Zuria district of Amhara, Ethiopia. We used a suite of nonexperimental mixed methods, including household trials of three distinct designs of playpens over several weeks, semi-structured interviews, structured direct observations, *Escherichia coli* testing of playpens and household floors, and a rudimentary consumer valuation exercise.

Central to the approach, known as trials of improved practices (TIPs),^27,28^ is consultation with the same informant-consultants over time to develop and test possible behavioral improvements, often involving a behavior-enabling product (in our case, playpens). We made three visits to each of the selected households for data collection. We collected data concurrently; then analyzed, interpreted, and reported all data sources together by research objective.

We also convened four group discussions immediately following the TIPs visits, which brought together study participant families, to compare all playpen models and discuss options for protecting their infants from exposure to animal excreta. To motivate the new behavior and assure the safety of playpen use, we conducted a combined playpen safety and behavior change promotion session at each household when the playpen was first introduced during TIPs Visit 1. The mother, as primary caregiver, was always present for this session, as were most husbands and other family members, because placement of the playpens was an attention-grabbing event. Participants in these sessions were counseled to clean their playpen and playmat whenever they appeared soiled (by urine, defecation, or dirt), and at least every 3 days. Between the TIPs Visits 2 and 3, health extension workers (HEWs) well-known in the study villages conducted a community-level behavior change session that included study participants and their husbands, to reinforce improved practices and encourage social support for playpen use and infant–animal separation. No data were collected from the community sessions.

**Figure 1.**
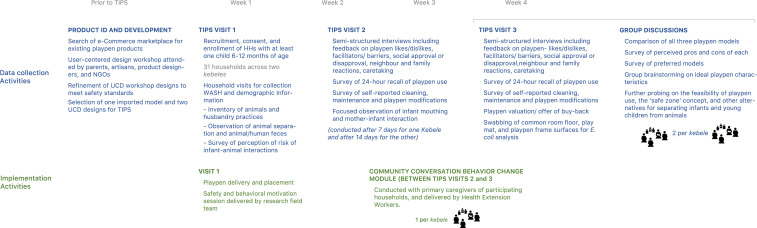
Study activities and methods. This figure appears in color at www.ajtmh.org.

The hypothesis of change integrated into this behavior change session was that playpen use and cleaning, together with other protective behaviors (e.g., sweeping compound to clear animal feces daily), are mostly determined by 1) perception of risk (of infant exposure to animals and feces), 2) self-efficacy and skills to create a safe zone for IYC, 3) access to an “enabling product” (the playpen), and 4) a supportive social environment. At the close of the session, caregivers and their husbands (if present) signed a certificate committing to adopt and maintain the protective behaviors introduced in the session. A safety reminder sheet also was left with members of each household, and both were tacked to the wall closest to the playpen as reminders of their public commitment. Figure 1 provides an overview of the study activities and methods.

### Playpen selection and development.

To identify playpens for testing, we searched in the domestic market for existing products with the potential to be affordable to rural Ethiopian households. We identified only a few imported “pack and play” options marketed to urban consumers which were clearly out of the financial reach of the rural poor. In parallel, we conducted an online search for playpen products manufactured outside Ethiopia that met U.S. Consumer Safety Standards^29^ (as Ethiopia has no safety standards for playpens). We identified a playpen model manufactured in China by North States brand, as potentially importable to Ethiopia for US dollar (USD) 16, inclusive of shipping, import duties, and taxes. We subsequently purchased North States playpens to serve as one of the three playpen models used in the study (referred to herein as “Model A”). We also codesigned and sourced playpens for household consumer testing using a user-centered design (UCD) approach, bringing together rural agrarian parents, HEWs from the Ethiopia’s maternal and child health program, and technical staff from the Ethiopian regional (Amhara) polytechnic college. The research team introduced UCD workshop participants to the animal waste risk problem, familiarized them with required safety standards and child development considerations, and challenged them to participate in an iterative design process using locally available materials and reflecting local user preferences. Two playpen models emerged for testing along with the imported North States model. We considered these models, which could be produced at USD 15 and 28, respectively, for materials and labor, to have the potential to be affordable to our target consumers if manufactured and sold at scale.

**Figure 2.**
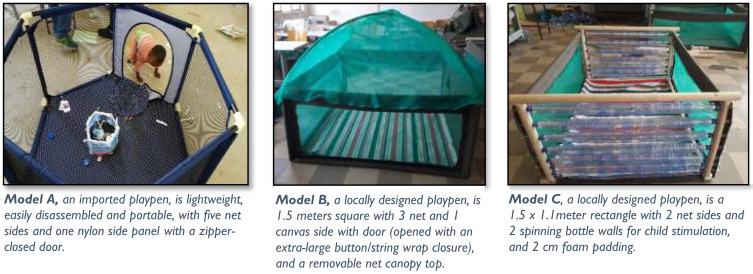
Playpen models included in trials of improved practices with main features. This figure appears in color at www.ajtmh.org.

We fabricated 12 units of each of the two playpens designed through the UCD workshop (referred to herein as “Model B” and “Model C”). All three models had a floor connected without a gap to the playpen walls and were fitted with a removable, cleanable playmat. We tested Models A and B in 10 households each, and Model C in 11 households (Figure 2).

### Site selection and sampling.

The 10 study villages were purposively selected using the following eligibility criteria:

1. High-to-medium access to water, defined as available within 30-minute roundtrip for collection and as classified by routine government management information systems (MIS)^30^ and the DHS, to assure the possibility of cleaning the playpen;
2. declared “open defecation free” (ODF) within the past 3 years, as tracked by the government MIS, to minimize human feces in the environment and build on demonstrated commitment to improving hygiene; and
3. less than 0.5 km or a 30-minute walk from a passable roadway, to facilitate placement of playpens and interviewer access.

Government health extension supervisors consulted their rosters and provided the names of all households in the villages with an infant aged 7–12 months and a caretaker aged at least 18 years. From these lists, we randomly selected up to six households from each village, for a total of 55 households meeting initial criteria. We added two additional eligibility criteria: engaging in subsistence agriculture and possessing at least three poultry and at least one cow, goat, or sheep. Members of the first three households per village who met the criteria were briefed, and the infant’s primary caregiver was invited to join the study, yielding a total of 30 households. Our sample included no female-headed households, and the mother was the primary caregiver in all cases. A fourth household inadvertently recruited in one village was retained in case a household dropped out of the study, bringing the total number of households to 31. For the four group discussions (two/*kebele*), we recruited 42 adult household members from all 31 study households to participate.

Our original protocol called for microbial sampling of playpens, playmats, and the common room floors in all study households, but shortages of analytical supplies necessitated the selection of a subsample of 23 of the 31 households for testing, with two swabs per household. We eliminated the most distant village in both *kebeles* (three households each) and randomly selected one additional household in each *kebele* for exclusion.

### Data collection.

Over a 4-week period in June and July of 2019, four experienced interviewers trained in study procedures made three home visits per household for the TIPs data collection. The interviewers collected data from the semi-structured interviews and observations with Android tablets using the SurveyCTO (Dobility Inc., Cambridge, MA) platform.^31^ They also took handwritten notes to capture responses not readily or fully covered by the pre-coded response categories. Detailed notes also were taken during each group discussion and were reviewed immediately afterward by the facilitator and notetakers as well as by one of the principal investigators (PIs).

### Data processing and analysis.

Interviewers were trained to code participant responses to open-ended questions directly into the corresponding set of pre-tested response categories. Any responses that interviewers could not clearly code into those categories were noted in as much detail as possible in the “other” category. Those responses were reviewed in regular team meetings and were entered into pre-coded response categories, if possible, or left as “other” for subsequent coding. Following quality control/quality assurance checks, we computed descriptive statistics using Statistical Package for Social Sciences and Microsoft Excel (Microsoft Corp., Redmond, WA).

Supplementary qualitative information collected during TIPs interviews was first documented in handwritten notes and subsequently transferred to Microsoft Word for coding. The two PIs assigned mostly a priori codes from the existing response categories, creating additional categories as needed, and then compared them for inter-coder reliability. We developed thematic matrices in Excel and incorporated the interview notes for further analysis.

Extensive notes from group discussions were reviewed by the discussion facilitator, the notetaker, and one of the PIs immediately following each discussion. Discussion notes were further reviewed, and thematic codes were confirmed by the facilitator and the PIs.

### Microbial testing.

We did not swab household surfaces at baseline or at Visit 2. However, all playpens and mats were thoroughly cleaned with 55% alcohol wipes after initial assembly and placement in households, to minimize possible external *E. coli* contamination through manufacture, transport, storage, and assembly. During TIPs Visit 3, we swabbed playpen rims, playmats, and common room floors for *E. coli.* Two swabs were taken from each of our subsample of 23 households. The first was one of the common room floors (taken 1.5 m from the doorway), in an area where infants were likely to crawl as part of their daily routines. The second was a composite swab of the playmat (taken in the center of the mat) and the playpen rim (sampled above the playpen door for playpen Models A and B; and swabbed on the side of the rim expected to have the most hand “traffic” for Model C, as it had no door). The composite was achieved by using each side of a single swab for the mat and playpen rim, respectively.

Precut templates demarcated the swabbing areas. Swabs were collected using a commercially available environmental sponge sampling kit, the EnviroMax Plus 6" Sterile Round Foam Swab and Collection Tube (Puritan Medical Products, Giulford, ME), which is a premoistened single swab with half neutralizing buffer and half 0.1% peptone water. After the swabbing, the sponges were returned aseptically to the tube. Tubes were sealed and transported in a thermal cool box to the regional laboratory in the town of Bahir Dar for microbiological analysis.

In the laboratory, we processed each sample as follows: 1) remove the sponge from the tube using sterile forceps, 2) add 7 mL of sterile water into the tube, 3) place the sponge back into the tube and seal it, 4) vortex the tube for 30 seconds, 5) incubate the tube for 5 minutes at room temperature, 6) vortex the tube for another 30 seconds, and 7) reopen the tube and pour the swab eluent into an empty 15-mL conical vessel. Steps were repeated, and the swab was pressed against the side of the tube to squeeze out as much remaining eluent as possible before the final wash solution was transferred to the 15-mL conical tube. A 1-mL aliquot of the eluent was then transferred to a Contact Dry^™^ EC presterilized plate containing culture medium and a cold water-soluble gelling agent (Hardy Diagnostics, Santa Maria, CA), recapped, and incubated at 35°C for 24 hours, and then counted. We report results as colony-forming units (CFUs) per centimeter square of the surface swabbed, with a maximum detection limit for this method at 4.26 CFU/cm^2^ (corresponding to 200 CFU/mL on the plate).

### Valuation/buyback offer.

At enrollment, study participants were told that they would be trying the playpen for a few weeks, and then returning it at the close of the study. They were also informed they would receive a small gift of appreciation for participation. After the household data collection was completed, we informed participants that we were leaving the playpen as the gift and noted their reactions. The participants were then offered a choice between keeping their playpen or receiving a payment of 500 Ethiopian birr (approximately USD 17 at the time), our preliminary estimate of an achievable consumer price of a playpen if imported or manufactured in-country from locally available materials at scale. A typical Ethiopian farmer earns about USD 100/month from selling agricultural products and/or labor, to provide a relative comparison of value (K. Tegenfeldt, USAID Mission Addis Ababa, personal communication, April 14, 2020).

## RESULTS

### Sociodemographic characteristics of respondents.

All 31 study respondents were the biological mothers and primary caregivers of the infants under study, ranging in age from 20 to 37 years, with a mean age of 27 years. Twenty-one of 31 caregivers reported never having attended school. Eight of the mothers had completed some primary education; only two had attended any secondary school. Additional data on respondents and their households are provided in Table 1.

**Table 1:** Characteristics of study participants and households

**Caregiver characteristics**	**(N=31)**
**Characteristic**	**% (n)**
**Age**	
20–24 years	33 (10)
25–29 years	33 (10)
30–34 years	26 (8)
35–39 years	9.7 (3)
**Education Level**	
Never in school	68 (21)
Primary (1–4)	13 (4)
Primary (5–8)	13 (4)
Secondary (9–12)	6.5 (2)
**Household Characteristics**	**(N=31)**
**Education Level (household)**	
Never in school	13 (4)
Religious/adult education	6.5 (2)
Primary (1–4)	39 (12)
Primary (5–8)	32 (10)
Secondary (9–12)	6.5 (2)
Diploma	3.2 (1)
**Household size**	
1–3	19 (6)
4–6	61 (19)
7–9	19 (6)
>9	0
**Animals in Household (average #)**	
Chickens	8.0
Cattle	3.0
Sheep	0.8
Dogs	0.6
Donkeys	0.4
Cats	0.4
Household cash-generating activities*	
Sell crops	61 (19)
Sell animal products	39 (12)
Petty trade	23 (7)
Sell labor	13 (4)
**Household possessions (as socio-economic proxies)**	
Cell phone	65 (20)
Electricity**	52 (16)
Functioning radio	23 (7)

* Total percentage adds up to greater than 100% as some respondents engage in multiple activities.

** One subdistrict containing five of the villages was electrified and the other not, which is reflected above. This was the only notable difference between the two subdistricts.

Study eligibility included household ownership of at least three poultry and one cow, sheep, or goat. Because of a recent chicken plague, one household accepted in the sample did not currently have chickens but was included in the sample because no other household in the village had children in or close to the 7- to 12-month age criteria; this household had several cattle and intended to restock their chickens in the near future. Households reported an average of eight chickens and three cattle per household, with an average of 0.8 sheep, 0.6 dogs, and 0.4 donkeys and cats across households.

### Water, sanitation, and hygiene access.

We selected households from ODF-declared *kebeles.* The teams documented no direct visible evidence of open defecation, but only 10 of 31 households owned a private latrine at the time of our visit. The latrines that we observed at the study households were pit latrines with low evidence of usage; only half appeared to be currently in use. None met minimum standards for an improved pit latrine, although one was well-maintained. We did not ask about defecation practices per se so did not report on open- and fixed-point defecation. However, given the lack of latrines in the community, the *kebeles* could not have been currently ODF, despite previous ODF certification.

Only two of the 10 latrines had fixed handwashing stations within 3 meters of the latrine, and one handwashing station was along the pathway back to the house. No handwashing materials (water, soap, and ash) were observed at any of the handwashing stations.

About half the respondent households (16 of 31) used a community borehole as their main source of water. Twelve accessed water from protected or unprotected springs, and three households resorted to standing ponds or gullies as their main water source.

### Caregivers’ playpen use patterns.

**Figure 3.**
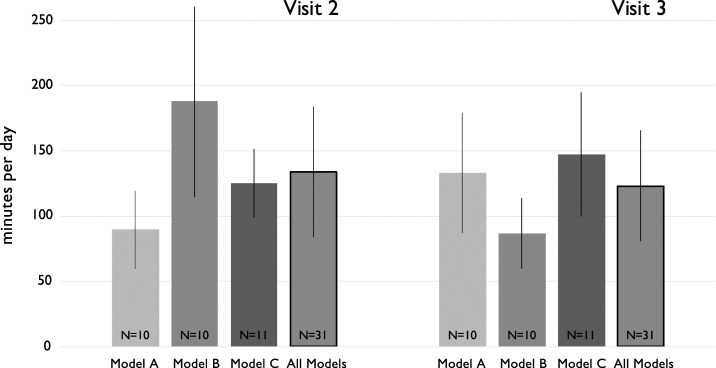
Mean reported time in playpen over past 24 hours by playpen model and visit. Error bars are SD, and sample sizes are noted within the columns.

We asked caregivers about playpen use during the previous 24 hours at both Visits 2 and 3. Combining all three playpen models, the average reported amount of time that infants spent in a playpen was 134 minutes/day at Visit 2 (SD = 100 minutes), and 123 minutes/day at Visit 3 (SD = 84 minutes) (Figure 3). We cannot reject the null hypothesis that the reported usage was equal between visits (*t*[60] = 0.45, *P* = 0.65). Disaggregating by playpen model, the only instance of a difference between visits large enough to be compatible with rejection of the null hypothesis of no change was for Model B (Visit 2 mean = 188 minutes/day, SD = 146; Visit 3 mean = 87 minutes/day, SD = 54), where (*t*[11] = 2.05, *P =* 0.065).

**Figure 4.**
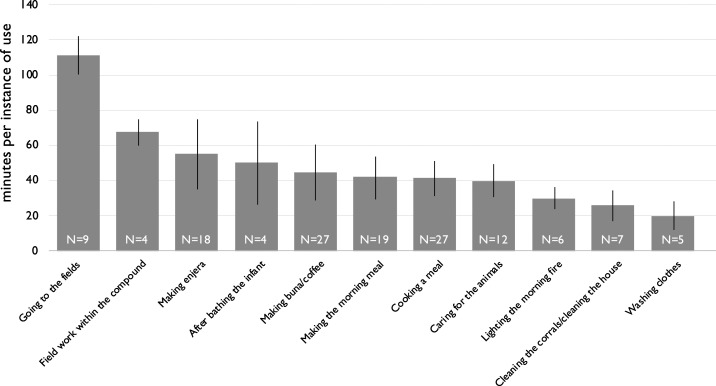
Reported duration of playpen time per instance of use, across Visits 2 and 3. Error bars are SD, and sample sizes are noted within the columns.

Combined over Visits 2 and 3, respondents reported placing infants in the playpen for 91 discrete instances of food preparation and cooking, 35 instances of household tasks (including cleaning and care of animals and their corrals as well as tending to garden plots within the compound), and nine instances of work outside the compound in the fields (with seven of nine instances occurring during Visit 3). The average duration of infant placement in the playpen ranged from 20 to 55 minutes for tasks within the compound (with the exception of tending to compound garden plots, whose four reported instances averaged 67 minutes). Instances of use of the playpen for tasks outside the compound, such as going to fields, were longer, averaging over 100 minutes (see Figure 4). In the two cases where respondents reported using the playpen when they went to the market, the durations were 20 and 180 minutes, respectively.

No nighttime use of the playpen was reported for any of the three playpen models through 24-hour recall, and only four of 31 caregivers (when asked directly about nighttime use) said that infants and other children slept in the playpen at night. Only the larger models (B and C) were used for nighttime sleeping.

When asked about their preferred occasions for putting an infant in the playpen, almost all caregivers during both Visit 2 and Visit 3 said the playpens were most useful to them when they were preparing food (60/61 or 98% combined over Visits 2 and 3) and cooking (56/61 or 92%). Other preferred occasions included when cleaning house and livestock corrals (43/61 or 56%) and when collecting water (23/61 or 38%). We also assessed whether households were using the playpens for the intended purpose of safely constraining the infant and using them according to the safety instructions. Because playpens were often not in use during interviews and observation, our direct observations of playpen use are limited, but in general, we observed the playpens (without infants) to be placed on stable ground, out of the path of smoke or bright sunlight, and at a safe distance from the household fire pit.

Many caregivers reported (and were observed) putting blankets or cloths over the playmats for cushioning, warmth, and/or to absorb urine, and some added pillows. Caregivers all reported liking the removable, smooth playmat, but some commented the plastic was cold for the infant. Although they had little hesitation putting infants into the playpens, they recognized a loss of body heat from their contact and from the typical leather baby carriers. As international safety guidance discourages use of sheets, blankets, or pillows because of risk of suffocation or strangulation, interviewers counseled against their use, encouraging instead that more clothing be put on infants. However, caregivers often insisted that these extra blanket-type items were essential.

### Changes in childcare practices with a playpen.

We asked caregivers about any shifts in childcare practices. Many (78%) reported that with a playpen, they were more comfortable leaving their infants with older children (while they collected wood and water), and 80% reported they were more confident watching their infants from a distance (for tasks such as preparing and cooking food). Similar patterns were recorded across the three models.

Meanwhile, the proportion of surveyed caregivers who reported bringing their infants to fetch water and firewood dropped after receipt of the playpen, from 58% (18 of 31) for both activities at Visit 1 to 33% (20/61) for water fetching and 36% (22/61) for wood fetching, combined over Visits 2 and 3. During these tasks, mothers usually left infants in the care of an older sibling.

Caregivers reported notable increases in their ability to watch infants while cooking, from 10 of 31 (32%) at Visit 1–22 of 31 (71%) at Visit 3. These reports are consistent with mothers reporting they found cooking and preparing food the most useful times for using the playpen, specifically because they could watch their infants from afar while the infants were in their playpens, at a safe distance from the fire. Daughters and sons were also reported to increasingly watch the infant during cooking, although at frequencies far lower than mothers; it is likely that infants in their playpens were being watched by one or multiple family members.

### Caregiver reactions to playpens.

When asked several questions about how caregiving changed with the playpen, as well as “good things” and “bad things” about using a playpen to watch the infant, mothers named a range of benefits to the infants’ hygiene, health, and motor development, as well as their own peace of mind and reduced physical and emotional burden.

At home interviews conducted at Visits 2 and 3, caregivers reported an array of perceived benefits of playpens, without meaningful differences between Visit 2 and Visit 3. Overall, 77% (47 of 61 responses) named protection from germs; 61% (37 of 61) named child safety; 48% (29 of 61) named prevention of child wandering; 40% (25 of 62) named child enjoyment of the space; and 25% (16 of 62) named the ability to help infants stand on their own. Interestingly, mothers mentioned without probing that their older children (also) loved to play in the playpen (23/62 responses or 37%). In the group discussions, many participants noted the playpens were useful to protect infants from wandering toward the fire and suffering burns, as well as keeping them cleaner, thus requiring less infant bathing and clothes washing.

Only three mothers found their infants would not tolerate being in the playpen. Two of these mothers had no older children, and both said it was difficult not having other children to help entertain their infants when they were in the playpens. Another mother who had just one child said she offered candy to neighbor children in return for them spending time in the playpen with her infant.

In addition to benefits to the infant, caregivers recognized benefits for themselves and their older daughters. Over Visits 2 and 3, 87% (54 of 62 responses) named “more time for chores” as a benefit; 79% (49 of 62) named “more freedom”; 66% (41 of 62) named not having to carry a child everywhere on their backs; 48% (28 of 61 responses) named “peace of mind”; and 39% (24 of 61) named being able to monitor their child from afar. In the group discussions, mothers said that now they have two arms to work with instead of one, elaborating that before it was as if they were handicapped, and now they were able to better attend to chores. (Ethiopian mothers often secure the child with a steady hand on the back, without the use of an infant carrier.)

Caregivers reported no negative outcomes from using the playpens, even when prompted by specific examples. They did note barriers to use and suggested improvements. Besides the three infants who would not tolerate being alone in the playpen, few child-related issues were mentioned as barriers. Rather, the barriers were primarily related to the physical characteristics of the playpen.

The most frequent suggestions for improvement were to add: 1) an absorbent covering on top of the plastic mat to absorb urine and provide some warmth, 2) padding under the mat or pen itself for cushioning (affecting comfort and to protect the infant from falls), and 3) an additional layer between the bottom canvas and dirt floor, to protect against moisture, condensation, and cow urine seeping into the canvas floor, which respondents mentioned would also prevent creating an environment for insects congregating and breeding. Many of the caregivers using Models A and C lamented the lack of a net covering to protect the infants from flies and mosquitos (whereas Model B included a net). Frequently, respondents also cited the lack of portability or difficulty in disassembling the playpens as a challenge to using them more, cleaning them easily, and taking them to the fields. We note, however, only two of the 10 households using the smaller, lighter, and easily disassembled Model A took their playpens to the fields. Another obstacle to taking any of the playpens to the fields was rainy weather, which created muddy conditions.

The design of the Model C playpen presented specific challenges. Two thick, wooden dowels of the playpen frame were exposed at interior ground level. A few caregivers recommended padding or covering the support frame. Some respondents, particularly those with the larger Model B, found the playpens too large for use in their common rooms. Caregivers appreciated that the larger model could accommodate more children but said that it was difficult to move around the house or bring outside.

### Playpen modifications.

We also assessed feasibility and appeal by monitoring any modifications to the playpens themselves or to their intended use. Just less than half of households at Visit 2 and just more than half of households at Visit 3 had modified their playpens to make them “easier” or “better” to use. Some caregivers with Models A or B added an absorbent cloth underneath the playpen floor to add padding between the child and the dirt floor, as well as to protect the underside of the canvas flooring from dirt and condensation. (Model C had a foam mattress on top of the canvas flooring [underneath the playmat], and no households with that model mentioned additional protection.) Two households expressed concern that Model C might become unstable, so they added reinforcing string ties to ensure the 5-cm detachable bars in the frame did not fall on the infant. (The only risk of a bar actually falling was if it were knocked by an older child climbing into the pen; the lack of a door in this model made it difficult for other children to enter.)

### Family and community responses to playpens.

Almost all caregivers reported that both family and community approved of the playpens. They reported their husbands, elders, infants’ older siblings, and nonrelatives were generally supportive and appreciative of the function of the playpens.

Many caregivers reported intense community interest and envy. (Almost all envy was reported as good-natured versus malicious.) Caregivers characterized neighbors as generally supportive and said many commented on what a good thing the playpen was. Others were curious to know how it worked and what was its function; caregivers reported they could confidently explain that a playpen helped create a safe zone for an infant.

### Items reported and observed in the playpens.

Interviewers found over 90% of playmats inside the playpens at both Visit 2 and Visit 3. Of the 31 playmats, two were found outside the playpen during a visit because they had been recently washed and were hanging to dry. Just one mat was being used independently. We observed no non-child–related uses of mats (such as for drying grains).

Most households had placed toys in the playpens to help stimulate their infants at both Visit 2 and Visit 3 (81% and 90%, respectively). Usually, the toy was the ball that had been offered with the playpen at Visit 1, but in some cases, another toy object (such as an empty plastic bottle) had been placed in the playpen to entertain the child. Different types of cloths, pillows, and other household objects were observed in the playpens in 29% of the households during both visits. We did not measure these objects for fecal contamination, but we note that any object in the playpen is a potential source of contamination.

During Visits 2 and 3, older children were often observed playing in the playpens without an infant, usually with another sibling other than the infant. Without exception, the older children had visibly soiled clothing and feet. The larger Model B was most often used by other siblings, with and without the infant, for play, napping, and eating. Model C had no door, so older children were lifted into it or, in a few cases, the playpen was placed close to a built-in bench along the wall to allow them to climb in. More than half of the infants were reported to have been playing with other children (mainly siblings) in the playpen. Not surprisingly, other children (and adults) were much more likely to be in the playpen in households that had the largest Model B playpen than those in households with the other, smaller models.

Households generally tolerated poultry on and inside the playpens, despite behavioral promotion to keep chickens away from the “safe zone” and out of the playpen. Chickens were observed roosting on two playpens, and poultry feces were observed on one playpen, with no effort on the part of the caretaker to remove them. Fresh cow feces were frequently seen within a half meter of the playpen and, on several occasions, were deposited there during a visit, only occasionally being removed (likely for future use rather than to distance the infant or playpen from the feces).

### Cleaning and maintenance of playpens.

At both Visit 2 and Visit 3, respondents were asked how the playpen had been cleaned and maintained “since the last visit” (over the past 7–14 days) and with what frequency. The majority of caregivers (74% during Visit 3) replied that they had washed the mat, and 58% reported that they had scrubbed the whole playpen. About half of the households (40% at Visit 2% and 60% during Visit 3) reported cleaning their playpens only when they looked dirty. Thirty percent of respondents at Visit 2 reported daily cleaning. At Visit 3, 10% of the respondents reported cleaning at least daily, and 30% reported cleaning “a few times” since the last visit.

### Microbial sampling results.

**Figure 5.**
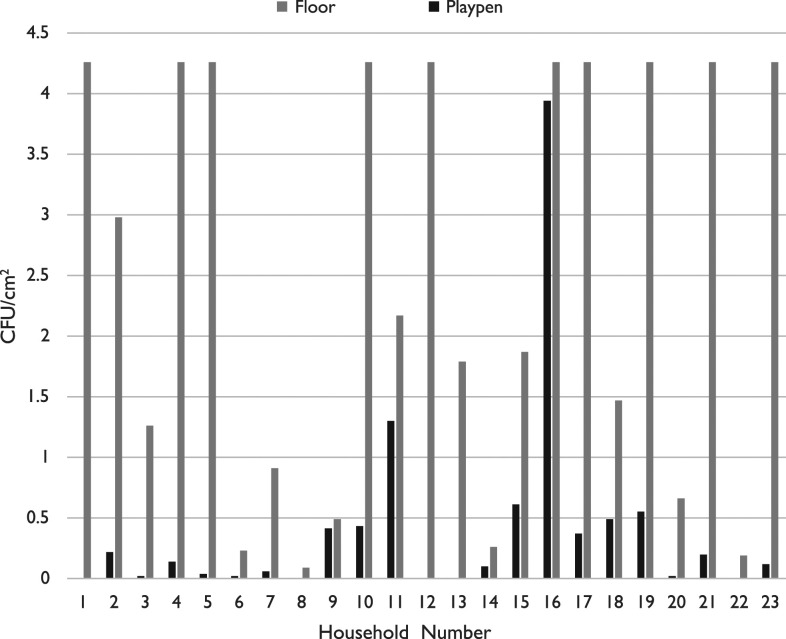
*Escherichia coli* counts in colony-forming units (CFU)/cm^2^, by density on floor and playpen/playmat composite.

*Escherichia coli* was detected in 18 of 23 playpen/playmat composite samples tested after 3 weeks in study households, but at densities far below those of floors sampled in the same household (Figure 5). Six playpens were contaminated at counts below 0.1 CFU/cm^2^, and all, but two, of the 23 playpens were < 1.0 CFU/cm^2^. By comparison, 10 of the 23 sampled floors were contaminated at the maximum detection limit of 4.26 CFU/cm^2^. (For reference, the Model C area totals 22,500 cm^2^, and the Model B area is 16,500 cm^2^.) We detected no relationship between the type of playpens and the contamination levels of the playpens.

### Valuation/buyback offer.

Respondents from all the participant households elected to keep the playpen rather than taking the relatively large cash payout. Five of the 31 caregivers first consulted with their husbands before deciding to keep the playpen, one consulted with her mother-in-law, and the remainder decided immediately. In general, all respondents were assessed to be quite happy to have this option.

## DISCUSSION

The health risks posed by IYC exposure to animal feces and exploratory mouthing are inadequately addressed by traditional WASH interventions. Our study explored the acceptability and feasibility of the use of playpens to reduce this generally previously underexplored risk to IYC health from ingestion of contaminated dirt and excreta itself.

Caregivers and their families found the physical playpens appealing and perceived myriad benefits. The concept of a safe zone resonated with caregivers and families and encouraged them to not only use the playpen itself but also to establish a clean, protected area dedicated to infants and not for animals that can be monitored and maintained. This is a key concept to take hold in these households, given the proliferation of animals and their feces in living spaces and the frequent lack of basic latrine and handwashing facilities on compounds, which likely contributed to a contaminated environment.^11,32,33^ It is possible that respondents’ reporting of benefits was biased by the emphasis on the benefits of playpen use during two motivational sessions (the first at Visit 1 and the second during a group session between Visits 2 and 3). However, because of the overall enthusiasm of respondents when reporting on the use of the playpen, bias is unlikely to have strongly influenced the nature or direction of the reporting.

We did not attempt to measure changes in IYC ingestion of dirt and feces, although caregivers did report perceived declines in those behaviors. However, there were other importantly reported benefits of playpen use, including reduced burden on caregivers (both mothers and young girls), less caregiver anxiety about infant safety, improved infant motor skills, infants staying cleaner, and reduced risk to infants of burns and exposure to indoor smoke. Nearly all respondents said that having the playpen made childcare easier.

Our results indicate that female siblings, and to a lesser extent male siblings, were tasked with watching the infants in the playpens when they previously may not have performed that task. However, the mothers’ reported perception was that the playpen eased the burden both for themselves and their older children, primarily because the infant was no longer on a caregiver’s back, so it was literally a reduced burden and figuratively freeing up the caregiver. Caregivers also commented that the playpens made movement easier and allowed caregivers to use both hands for tasks. In addition, they felt that the playpens served to limit risk to infants while they were left in the care of an older sibling, particularly the risks of burns, precipitous falls, contact with sharp objects, or wandering into an unknown or unsafe space (which included proximity to large livestock). For older siblings, who often joined the infants in the playpens, there were indications that infant care could be considered fun, rather than a burden. The playpens also enabled the girl-child caretakers to supervise and engage infants from a (near) distance. Mothers remarked that “*my [older] daughter is now free to read,”* and *“[she] can now focus on homework and play*.” All respondents chose to keep the playpens, rather than accepting the cash equivalent of a price potentially viable for a scaled up commercial playpen enterprise.

Both the playpens and the concept of a safe zone for IYC were also acceptable and appealing to the broader community. Local leadership endorsed the playpen concept after participating in an initial community session and continued to support the trial. Neighbors who stopped by the participant households while interviewers were visiting commented positively on the novel playpen. The only negative reactions reported by study participants were that some community members, HEWs, and local officials were disappointed that their households were not included in the study and thus did not have an opportunity to try out the innovation of a playpen.

Because utilization of the playpens was universal among study participants, we were unable to compare users and nonusers and, therefore, cannot report which determinants drove behavior. We can only offer insights based on reported enablers and barriers. The mesh sides that enabled caregivers to see the infants and supervise them from a distance were often named as enhancing feasibility of use, as were the mosquito net of Model B and the soft foam mat and built-in play of Model C. All, but three, mothers reported their infants were content in the playpens. However, many said that their infants required entertainment, particularly an older sibling to keep them company, while in the playpen. Mothers who had older children did not consider this a problem because the older siblings appeared to enjoy the activity, and it freed up the mother to more efficiently perform her daily tasks.

Moderate cleaning of playpens was reported, with about half of households at both visits 2 and 3 reporting thorough cleaning of the entire playpen “whenever it looked dirty.” Respondents reported appreciation that the washable material and the portability of the removable playmat facilitated its cleaning. Some respondents seemed unconcerned about the need to routinely clean their playpens in the absence of visible dirt. Although our bacteria sampling indicate that playpens were much less contaminated than the household floor where the children otherwise played, *E. coli* levels reflected usage over just a 3-week period; contamination might continue to increase over time. (e.g., contamination levels of floors reflect accumulation over a longer period because we did not clean dirt floors at baseline.) Some households mentioned long distances to water supply sources limiting their ability to clean the playpens and mats.

All, but one, of the 31 study households reported using the playpens daily. However, despite interview responses reflecting feasibility of use and product appeal, caregivers reported keeping young children in the playpens only for an average of about 2 hours/day.

A TIPs study of a larger “community” play yard in Zambia found many similar findings. Self-reports from caregivers suggested that a large, community-built play yard protected IYC from ingesting soil and livestock feces. Use was reported differently than in this Ethiopia study; Reid et al.’s^25^ study did not calculate total time reported in the playpen each 24-hour recall but did document total episodes of playpen use per day, which were similar counts to those reported in our 24-hour recalls. The range of perceived benefits of play yards in the Zambia study was similar to that we reported here for Ethiopia, but community reactions to the play yards in Zambia were more negative and presented a barrier to household use. In addition, the Zambia study also did not document shifts in caretaking with playpens but did conclude that more research is needed “to examine the role of women’s time of use in their home environment, community reactions to the intervention, and the biological efficacy to reduce microbial ingestion.” In an evaluation of interventions designed to reduce childhood drowning in Bangladesh, actual time in the playpen was not reported, but the authors described compliance as extremely low because children aged 12–47 months were reported to not want to stay in the playpens.^23^ The authors do not explicitly discuss the relationship of age and willingness to stay in the playpen, but given the high mobility and independence of 3- to 4-year-olds (the upper age range of IYC in the study), resistance to extended stays in a playpen can be expected. No international standards exist about recommended time in playpens, in part because the key child development concern is not time spent in a playpen per se, but assuring responsive parenting, supervision, stimulation, and an age-appropriate amount of independence and engagement.

One factor that limited extended use of the playpen was that all the infants in our study were actively breastfeeding and often wanted to return to their mothers for feeding and comfort. Lack of portability to bring the playpens outside and to the fields also limited feasibility of use, particularly later in the study when most family members (including children) were assisting with planting. However, even the most portable model (Model A) was used in the fields on only a few occasions, suggesting that other factors, including muddy paths and fields and perhaps habit, played a larger role in limiting use.

A final consideration potentially affecting reported time in the playpens was the difficulty many respondents had with estimating time intervals when they were led through 24-hour recall. To address this challenge, interviewers were trained to guide estimations based on familiar intervals, such as the time required to reach the road or to make *shiro* (a common traditional food of ground legume, which takes about 30 minutes to prepare). This approach provided some standardization in the responses regarding time intervals. We do not know how that approach addressed this limitation, and the degree to which people over- or underestimated time spent in the playpens. Other techniques for estimating time more accurately, such as installing a motion sensor video camera, were considered but not pursued because they would have introduced a host of ethics and privacy issues.

Separating IYC from animals was not a new concept to the study participants because it is stressed as part of the national maternal and child health package and HEWs’ messages to households; still, it is one they felt was challenging to achieve in their agricultural environment, where animals and humans intermingle. Findings from this study may help enhance outreach worker appeals to parents, but determination of actual exposure reductions from playpen use would better inform broader policy recommendations.

In both household interviews and group discussions, many participants repeated their commitments to use their playpens to prevent children from eating dirt and feces. Nevertheless, we observed risky practices that could expose IYC to feces in households, such as allowing poultry on and inside the playpens as well as multiple older children with visibly soiled feet and clothing. Older children used the playpen for their own entertainment in the course of watching an infant, and even adults napped in the large playpen with the mosquito net. The net feature and the cool, smooth playmat were appealing to children and adults alike and may well have brought contamination into the playpens. Not surprisingly, many playpens exhibited some *E. coli* contamination even within the 3-week period of study*.*

One limitation of our study is that we examined only short-term use, acceptability, and feasibility of the playpens. We did not measure how much time the study infants spent on dirt floors nor how much time infants without a playpen option spend on dirt floors. Little is known about thresholds of animal excreta and contaminated soil exposure for effects on health and child growth. One large randomized controlled trial that included a playpen component and began to address the animal source/mouthing vector succeeded in reducing exploratory mouthing but did not result in improvements in infant health or growth.^20^ In our study, we cannot conclude whether the time the children spent on the comparatively cleaner surfaces of the playpens resulted in a sufficiently lower exposure to pathogens to result in any measurable health or growth benefits. Another key limitation of our TIPs research is the small sample size and short study duration, particularly as it is divided across three playpen models over roughly 1 month. This (in addition to unique features of the Ethiopian context) limits generalizability. In addition, the relatively short interval of use in households limited documentation of use as the infants aged.

Based on the results of this study, we cannot conclude that playpens alone plausibly protect IYC from environmental contamination. Promotion of playpen use would certainly need to be part of a more comprehensive effort to maintain a hygienic environment, but this study did demonstrate that access to the enabling technology of a playpen, together with promotion of a safe zone, bolstered self-efficacy and intention to reduce infant exposure to pathogens.

We identified a number of other perceived benefits of playpen use for caregivers, IYC, and siblings that are noteworthy. These additional benefits include reduced burden on women, with possible impact on their mental health, reduced burden on young girl caretakers, and other possible health benefits, such as reduced exposure to cooking emissions that are tied to childhood pneumonia^34^ and childhood stunting and related impaired cognitive development of IYC.^35–37^ Our results support further exploration of the generalizability of these findings as well as the potential benefits and commercial viability of scaling up use of playpens in rural, agricultural households as part of a comprehensive approach to IYC development and women’s empowerment.
